# Supplementary material for: 13C, 25Mg, and 43Ca Solid-State NMR for the Purpose of Dolomitic Marbles Provenance Elucidation
Source: Materials (Basel). 2023 Feb 9;16(4):1468. doi: 10.3390/ma16041468 (PMC9960183; doi:10.3390/ma16041468)
Supplement: Supplementary file 1 [file materials-16-01468-s001.zip › materials-2000043-supplementary.pdf]

materials

MDPI

Supplementary Material

^{13}C, ^{25}Mg, and ^{43}Ca Solid-State NMR for the Purpose of Dolomitic Marbles Provenance Elucidation

Isabelle Pianet [1,*], Anna Gutiérrez Garcia-Moreno [2], Marie-Claire Savin [2,3], Nicolas Frerebeau [1], Julien Trebosc [4], Pierre Florian [5] and M. Pilar Lapuente Mercadal [2,6]

1 Department of Archaeology, Archéosciences Bordeaux, UMR CNRS-Université Bordeaux-Montaigne 6034, Maison de l'archéologie, Esplanade des Antilles, 33600 Pessac, France; nicolas.frerebeau@u-bordeaux-montaigne.fr
2 Unitat d'Estudis Arqueomètrics, Institut Català d'Arqueologia Clàssica, Plaça d'en Rovellat, 43003 Tarragona, Spain; agutierrez@icac.cat (A.G.G.-M.); marieclaire.savin@uab.cat (M.-C.S.); plapuent@unizar.es (M.P.L.M.)
3 Departament de Ciències de l'Antiguitat i de l'Edat Mitjana, Universitat Autònoma de Barcelona, Cerdanyola del Vallès, 08193 Barcelona, Spain
4 Department of Chemistry, Institut Michel Eugène Chevreul, CNRS FR 2638, Université Lille, Avenue Paul Langevin, 59655 Villeneuve d'Ascq, France; julien.trebosc@univ-lille.fr
5 Department of Chemistry, Conditions Extrêmes et Matériaux: Haute Température et Irradiation UPR CNRS 3079, 1D Avenue de la Recherche Scientifique, 45071 Orléans, France; pierre.florian@cnrs-orleans.fr
6 Department of Earth Sciences, Petrology and Geochemistry, University of Zaragoza (UNIZAR), C/Pedro Cerbuna, 12, 50009 Zaragoza, Spain
* Correspondence: isabelle.pianet@u-bordeaux-montaigne.fr

Table S1. Summary of samples analysed. Geologic dolomitic marbles TH-V are coming from the Cape Vathy quarry located in Thasos Island (Greece). CN en Mi from Coin and Mijas locatic in the Betic chain (Spain). ST from the district of Lez-Saint-Béat in the Pyrénées (France). Archeologic samples labelled MM are coming from the archeologic museum of Malaga and were taken from the Menula Victory (6 MM). an Aquatic divinity (24 MM) and a Male figure (29 MM) and from the antic theatre of Caesaraugusta (Zaragoza, Spain) where CL is a fragment taken from a decorative plate and BL from a moulding. Lapuente et al. 2002 [1], Lazzarini et al. 1980 [2], Lapuente 2022 [3],] Blanc & Lapuente 2020 [4] and Lapuente et al. 2009 [5].

Sample	Origin	Quantitative CL	Petrography	Isotopes $^{13}C/^{18}O$	Elemental Analysis	NMR ^{13}C	NMR ^{43}Ca	NMR ^{25}Mg
TH-V1	Thasos	[2]	[5]	[5]	here	here	nm	nm
TH-V2	Thasos	[2]	[5]	[5]	here	here	nm	nm
TH-V3	Thasos	[2]	[5]	[5]	here	here	nm	nm
TH-V5	Thasos	[2]	[5]	[5]	here	here	nm	nm
TH-V6	Thasos	[2]	[5]	[5]	here	here	nm	nm
TH-V7	Thasos	[2]	[5]	[5]	here	here	here	here
CN1	Coín	[4]	[1]	[1]	here	here	here	here
CN2	Coín	[4]	[1]	[1]	here	here	nm	nm
CN3	Coín	[4]	[1]	[1]	here	here	nm	nm
CN4	Coín	[4]	[1]	[1]	here	here	nm	nm
Mi1	Mijas	[4]	[1]	[1]	here	here	nm	nm
Mi8	Mijas	[4]	[1]	[1]	here	here	nm	nm
Mi10	Mijas	[4]	[1]	[1]	here	here	nm	nm
Mi16	Mijas	[4]	[1]	[1]	here	here	nm	nm
ST110	Lez-St-Béat	[3]	[3]	[3]	here	here	here	here
ST106	Lez-St-Béat	[3]	[3]	[3]	here	here	nm	nm
ST117	Lez-St-Béat	[3]	[3]	[3]	here	here	nm	nm
ST102	Lez-St-Béat	[3]	[3]	[3]	here	here	nm	nm

L3	Lez-St-Béat	-	-	-	here	here	nm	nm
L4	Lez-St-Béat	-	-	-	here	here	nm	nm
L7	Lez-St-Béat	-	-	-	here	here	nm	nm
6 MM	Málaga Museum	[1]	[1]	[1]	here	here	nm	nm
24 MM	Málaga Museum	[1]	[1]	[1]	here	here	nm	nm
29 MM	Málaga Museum	[1]	[1]	[1]	here	here	here	here
CL	Caesar Augusta	[5]	[5]	nm	here	here	nm	nm
BL	Caesar Augusta	[5]	[5]	[5]	here	here	here	here

*here—the samples are treated in the present paper; nm—not measured; Quantitative CL—Quantitative Cathodo Luminescence

Table S2. Nuclides observed by NMR spectroscopy and their characteristics.

Isotope	Natural Abundance (%)	Nuclear Spin (I)	Magnetogyric Ratio ($\gamma/10^7$, rad $T^{-1}s^{-1}$)	Quadruple Moment (10^{28} Q/m^2)	Resonance Frequency (MHz) at 20T (850 MHz for ^1H)	Relative Sensitivity (^1H = 1.00)	Absolute Sensitivity (^1H = 1.00)
^{43}Ca	0.135	7/2	−1.8025	0.2	57.296	6.4×10^{-3}	9.28×10^{-6}
^{25}Mg	10.13	5/2	−1.639	0.22	52.106	2.67×10^{-3}	2.71×10^{-4}
^{13}C	1.108	1/2	6.7283	0	212.875	1.59×10^{-2}	1.76×10^{-4}

Table S3. Physico-chemical parameters extracted from ^{13}C NMR Spectra of quarries and artefacts of all the samples studied. δ corresponds to the NMR ^{13}C chemical shift with respect to tetramethyl silane which resonates at 0 ppm; area is the integration of the area of the signal with respect to an external reference; $\Delta\nu_{1/2}$ is the full width at half-maximum of the carbonate resonance.

Sample N°	Quarry	Area	NMR	
			Δν (Hz)	δ (ppm)
24,790	Thasos	1.02	80	170.8
24,791	Thasos	1.10	80	170.81
24,792	Thasos	2.13	109	170.79
24,793	Thasos	0.74	73	170.79
24,794	Thasos	1.24	87	170.8
24,795	Thasos	1.70	94	170.83
24,796	Thasos	0.83	73	170.77
	mean	1.25	85.14	170.80
	Std	0.50	12.90	0.02
24,782	Coin	0.36	58	170.78
24,783	Coin	1.06	80	170.79
24,784	Coin	0.57	73	170.8
24,785	Coin	0.54	65	170.84
	mean	0.63	69.00	170.80
	Std	0.30	9.56	0.03
24,786	Mijas	1.08	80	170.8
24,787	Mijas	1.57	73	170.82

24,788	Mijas	2.13	102	170.82
24,789	Mijas	2.10	102	170.82
	mean	1.72	89.25	170.82
	std	0.50	15.00	0.01
24,778	Lez-Saint-Béat	1.01	80	170.78
24,779	Lez-Saint-Béat	1.62	80	170.77
24,780	Lez-Saint-Béat	1.54	80	170.81
24,781	Lez-Saint-Béat	0.98	73	170.82
22,169	Lez-Saint-Béat	1.80	83	170.74
22,168	Lez-Saint-Béat	1.80	80	170.75
	mean	1.46	79.33	170.78
	std	0.37	3.33	0.03
6 MM	item	0.99	80	170.81
24 MM	item	1.80	102	170.79
29 MM	item	0.75	65	170.81
CL	item	1.22	80	170.79
BL	item	1.32	80	170.76

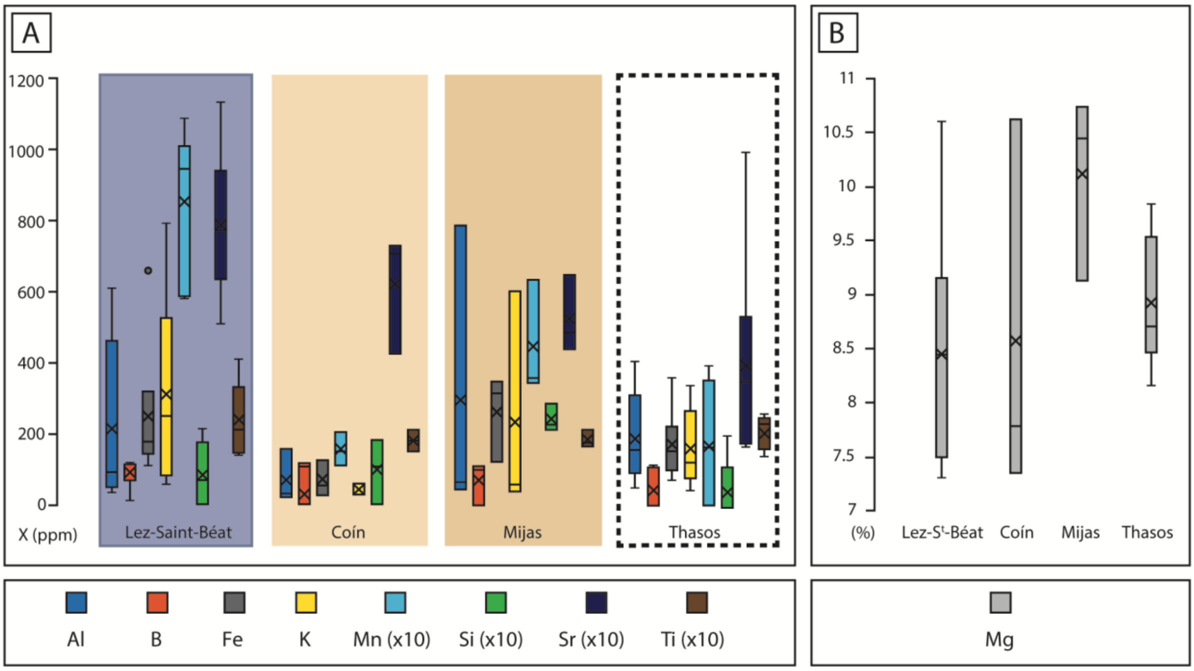

Figure S1. Composition on different elements depending on the quarry. (**A**). boxplot for X = Al, B, Fe, K in ppm. and Mn, Si, Sr, Ti in ppm × 10. (**B**). Boxplot for Mg in %.

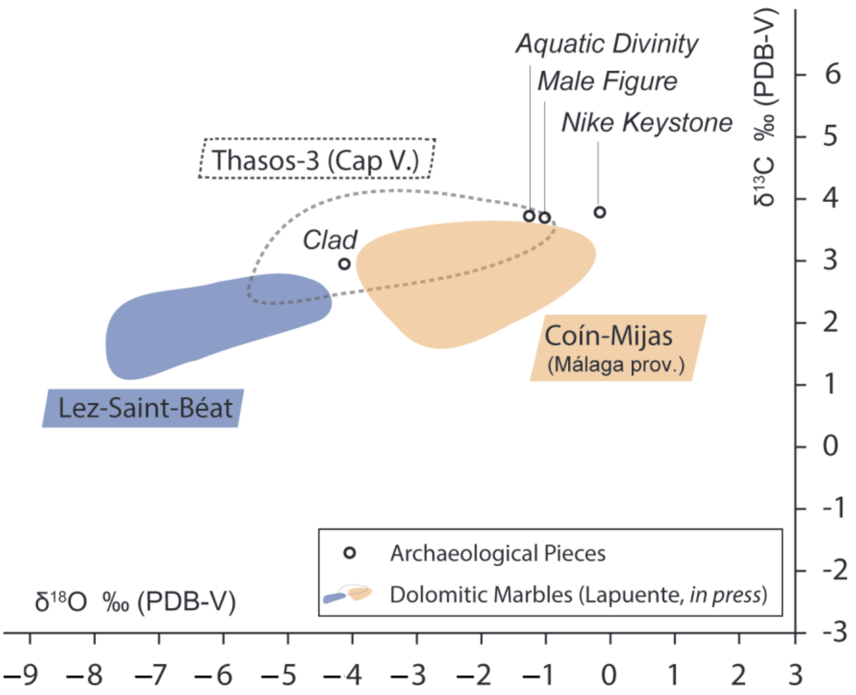

Figure S2. δ ^{13}C vs ^{18}O diagram of dolomitic marbles. Values are data collected from [1–3]. The designation of Coín-Mijas (Málaga) has been used because the δ ^{13}C and δ ^{18}O values of the two quarries of Coín and Mijas (in the district of Málaga) are too close to be distinguished.

References

1. Lapuente Mercadal, P.; Martinez, M.P.; Turi, B.; Blanc, P. Characterization of Dolomitic Marbles from the Malaga Province (Spain). In *Interdisciplinary Studies on Ancient Stone: Proceedings of the Fifth International Conference of the Association for the Study of Marble and Other Stones in Antiquity*, Museum of Fine Arts: Boston, MA, USA, 2002; pp. 152–162.
2. Lazzarini, L.; Mariottini, M. La provenienza dei marmi cristallini usati in antico: Un nuovo contributo al problema del rapport calcite/dolomite. *Bollettino d'arte* **1987**, *41*, 69–72.
3. Lapuente Mercadal, M.P. El Pirineo, una fuente principal de mármol en el occidente romano. In *Poder y prestigio en mármol. Homenaje a Isabel Rodà de Llanza*.; Gorostidi del Pi, D.; Gutiérrez Garcia-M., A.; Eds.; Anejos de Archivo Español de Arqueología; Consejo Superior de Investigaciones Científicas: Madrid, Spain, 2022; Vol. XCIV, pp. 409–429.
4. Blanc, P.; Lapuente Mercadal, M.P.; Gutiérrez Garcia-M., A. A New Database of the Quantitative Cathodoluminescence of the Main Quarry Marbles Used in Antiquity. *Minerals* **2020**, *10*, 381
5. Lapuente, P.; Turi, B.; Blanc, P. Marbles and Coloured Stones from the Theatre of Caesaraugusta (Hispania): Preliminary Study. *In Proceedings of the 7th International Conference of Association for the Study of Marble and Other Stones in Antiquity*, Thasos, Greece, 15–20 September 2003, Ed.; Maniatis, Y.; Ecole française d'Athènes: Athens, Greece, 2009; 509–522.
